# ASCancer Atlas: a comprehensive knowledgebase of alternative splicing in human cancers

**DOI:** 10.1093/nar/gkac955

**Published:** 2022-11-01

**Authors:** Song Wu, Yue Huang, Mochen Zhang, Zheng Gong, Guoliang Wang, Xinchang Zheng, Wenting Zong, Wei Zhao, Peiqi Xing, Rujiao Li, Zhaoqi Liu, Yiming Bao

**Affiliations:** National Genomics Data Center & CAS Key Laboratory of Genome Sciences and Information, Beijing Institute of Genomics, Chinese Academy of Sciences and China National Center for Bioinformation, Beijing 100101, China; University of Chinese Academy of Sciences, Beijing 100049, China; CAS Key Laboratory of Genomic and Precision Medicine, Beijing Institute of Genomics, Chinese Academy of Sciences and China National Center for Bioinformation, Beijing 100101, China; University of Chinese Academy of Sciences, Beijing 100049, China; National Genomics Data Center & CAS Key Laboratory of Genome Sciences and Information, Beijing Institute of Genomics, Chinese Academy of Sciences and China National Center for Bioinformation, Beijing 100101, China; University of Chinese Academy of Sciences, Beijing 100049, China; National Genomics Data Center & CAS Key Laboratory of Genome Sciences and Information, Beijing Institute of Genomics, Chinese Academy of Sciences and China National Center for Bioinformation, Beijing 100101, China; University of Chinese Academy of Sciences, Beijing 100049, China; National Genomics Data Center & CAS Key Laboratory of Genome Sciences and Information, Beijing Institute of Genomics, Chinese Academy of Sciences and China National Center for Bioinformation, Beijing 100101, China; University of Chinese Academy of Sciences, Beijing 100049, China; National Genomics Data Center & CAS Key Laboratory of Genome Sciences and Information, Beijing Institute of Genomics, Chinese Academy of Sciences and China National Center for Bioinformation, Beijing 100101, China; National Genomics Data Center & CAS Key Laboratory of Genome Sciences and Information, Beijing Institute of Genomics, Chinese Academy of Sciences and China National Center for Bioinformation, Beijing 100101, China; University of Chinese Academy of Sciences, Beijing 100049, China; National Genomics Data Center & CAS Key Laboratory of Genome Sciences and Information, Beijing Institute of Genomics, Chinese Academy of Sciences and China National Center for Bioinformation, Beijing 100101, China; University of Chinese Academy of Sciences, Beijing 100049, China; CAS Key Laboratory of Genomic and Precision Medicine, Beijing Institute of Genomics, Chinese Academy of Sciences and China National Center for Bioinformation, Beijing 100101, China; National Genomics Data Center & CAS Key Laboratory of Genome Sciences and Information, Beijing Institute of Genomics, Chinese Academy of Sciences and China National Center for Bioinformation, Beijing 100101, China; University of Chinese Academy of Sciences, Beijing 100049, China; CAS Key Laboratory of Genomic and Precision Medicine, Beijing Institute of Genomics, Chinese Academy of Sciences and China National Center for Bioinformation, Beijing 100101, China; University of Chinese Academy of Sciences, Beijing 100049, China; National Genomics Data Center & CAS Key Laboratory of Genome Sciences and Information, Beijing Institute of Genomics, Chinese Academy of Sciences and China National Center for Bioinformation, Beijing 100101, China; University of Chinese Academy of Sciences, Beijing 100049, China

## Abstract

Alternative splicing (AS) is a fundamental process that governs almost all aspects of cellular functions, and dysregulation in this process has been implicated in tumor initiation, progression and treatment resistance. With accumulating studies of carcinogenic mis-splicing in cancers, there is an urgent demand to integrate cancer-associated splicing changes to better understand their internal cross-talks and functional consequences from a global view. However, a resource of key functional AS events in human cancers is still lacking. To fill the gap, we developed ASCancer Atlas (https://ngdc.cncb.ac.cn/ascancer), a comprehensive knowledgebase of aberrant splicing in human cancers. Compared to extant databases, ASCancer Atlas features a high-confidence collection of 2006 cancer-associated splicing events experimentally proved to promote tumorigenesis, a systematic splicing regulatory network, and a suit of multi-scale online analysis tools. For each event, we manually curated the functional axis including upstream splicing regulators, splicing event annotations, downstream oncogenic effects, and possible therapeutic strategies. ASCancer Atlas also houses about 2 million computationally putative splicing events. Additionally, a user-friendly web interface was built to enable users to easily browse, search, visualize, analyze, and download all splicing events. Overall, ASCancer Atlas provides a unique resource to study the functional roles of splicing dysregulation in human cancers.

## INTRODUCTION

Pre-mRNA splicing is a fundamental process in mammalian gene expression, with nearly 95% of human genes undergoing alternative splicing (AS) to generate protein diversity (1). Over the past decade, there have been extensive evidences showing that aberrations in mRNA splicing contribute to neoplastic transformation, tumor progression and therapeutic resistance (2). Splicing dysregulation can alter a multitude of critical cellular processes, covering all cancer hallmarks (3). For instance, AS of *PKM* regulates tumor metabolism (4), the splicing variants of *CD44* contribute to epithelial to mesenchymal transition (5), pre-mRNA splicing of *BCL2L1* or *MCL1* induces apoptosis (6), the splicing variant *RAC1b* is associated with cell growth (7). The recent large-scale genomic analysis has revealed the mutational landscape of splicing-related genes in human cancers, which provides genetic evidence directly linking RNA splicing dysregulation to cancers (8). Moreover, a comprehensive pan-cancer study showed that tumors harbor up to 30% more splicing events and potential neo-junctions versus normal tissues (9). These analyses highlight the pervasive existence of pathogenic splicing changes in human cancers.

To date, several useful cancer splicing resources have been established with different purposes. For example, OncoSplicing (http://www.oncosplicing.com/) aims to provide clinically relevant alternative splicing events (10). LncAS2Cancer (https://lncrna2as.cd120.com/) mainly focuses on alternative splicing of lncRNAs (11). ExonSkipDB is a functional annotation database only about exon skipping events (12). CancerSplicingQTL is designed to identify potential splicing quantitative trait loci (13). All these databases were built based on AS events identified from computational tools by analyzing cancer transcriptome datasets. Although computationally extensive, only a small subset of these events has been successfully validated experimentally. In addition, simply a tiny subset of the validated events is crucial for tumorigenesis. It remains a great challenge to extract AS events functionally relevant to cancers from the lengthy list of computational predictions. Meanwhile, the archival of cancer-associated AS events from the tremendous efforts of mechanism studies is of great necessity to systematically understand the regulation of altered splicing events and their associations in cancers. Unfortunately, there is still a lack of such resources, which serve as not only a collection of computationally putative AS events for correlation-based analysis, but more importantly, a knowledgebase of key functional AS events together with its upstream splicing regulators, downstream oncogenic effects and possible therapeutic strategies.

**Figure 1.**
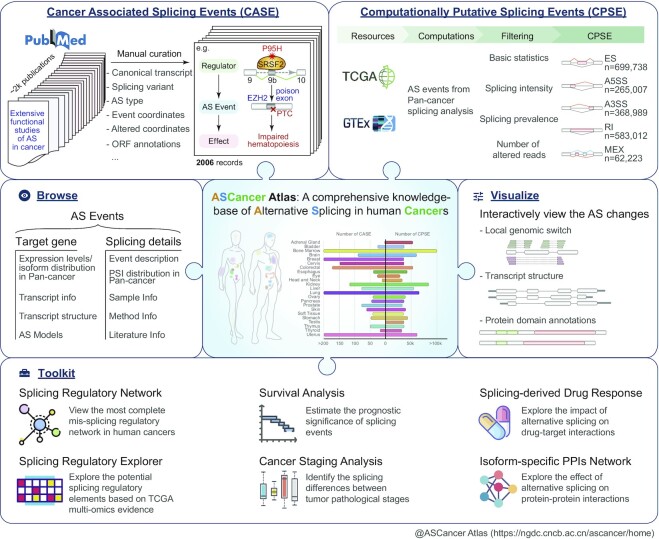
Schematic overview of ASCancer Atlas. It mainly includes two categories of splicing events: CASE and CPSE. In addition, a user-friendly web interface is provided where users can browse, search, visualize, analyze and download all splicing events of interest. Abbreviations: AS: alternative splicing. PTC: premature termination codon. ES: exon skipping. IR: Intron retention. A3SS: alternative 3′ splice site. A5SS: alternative 5′ splice site. MEX: mutually exclusive exons.

To fill this gap, we present ASCancer Atlas, a comprehensive knowledgebase of alternative splicing in human cancers for browsing, searching, visualizing, analyzing and downloading cancer-associated splicing events (CASE), as well as computationally putative splicing events (CPSE). In the current release, a total of 2006 high-confidence CASE were manually curated from 610 publications. These CASE have a unified curation model, including upstream splicing regulations, splicing event annotations, downstream oncogenic effects, and possible treatments. Meanwhile, ASCancer Atlas houses about 2 million CPSE, covering 33 TCGA (14) cancer types and 31 GTEx (15) normal tissues. In addition, an interactive splicing visualization tool and a multi-dimensional online analysis toolkit were equipped to further explore the splicing events. Collectively, ASCancer Atlas provides the first knowledgebase of carcinogenic AS in human cancers, which can help users to investigate the full spectrum of splicing dysregulation in cancers and will become a value-added resource when more and more splicing variants are identified as targets for cancer treatment. The overall design of ASCancer Atlas is shown in Figure 1.

## DATA COLLECTION AND PROCESSING

### Cancer-associated splicing events curation

To provide high-confidence cancer-associated splicing events, ASCancer Atlas set up a standardized and stringent curation process, mainly involving literature searching and filtering, as well as information retrieval and annotations. Firstly, we screened about 2000 literatures in PubMed (16) using the search term (‘alternative splicing’ OR ‘splicing variant’) AND (‘cancer’ OR ‘tumor’), and picked out the cancer-associated splicing events validated by experimental methods, such as RT-PCR and Western blot. Secondly, we manually curated the complete functional axis of mis-splicing events, including upstream splicing regulations, aberrant splicing events, downstream oncogenic effects, and potential therapeutic interventions. Lastly, the key information of each study was also recorded, such as genomic coordinates, transcript IDs, ORF annotations, cancer types/cell lines, experimental primers, and publication details.

### Computationally putative splicing events mining

The computationally putative splicing events were mined from the pan-cancer atlas project of alternative splicing analysis of 8705 tumor patients across 33 cancer types and 31 normal tissues (https://gdc.cancer.gov/about-data/publications/PanCanAtlas-Splicing-2018) (9). ASCancer Atlas used a series of filtering criteria referred to other useful cancer splicing resources (9,10), including three main steps: (i) keep the AS events, which have available PSI values for more than one-third of the cohort; (ii) keep the AS events with median PSI value >0.1; (iii) keep the AS events with at least 10 reads supporting the event.

In order to further identify the cancer-specific splicing events, differential AS analysis was performed between disease and normal samples. As most TCGA cancer types lack enough matched normal controls, we took an alternative way to compare the splicing differences between TCGA tumors and GTEx samples of the corresponding tissue types. Differential AS analysis was performed for TCGA cancer types with at least 30 tumor samples and 10 adjacent normal samples or at least 10 normal samples in the corresponding GTEx tissue types. The Wilcox rank sum test was used to evaluate the significance of the difference. The splicing events with |delta PSI| ≥ 0.1 and BH adjusted *P*-value ≤ 0.05 were selected as cancer-specific events.

### Splicing events visualization and splicing analysis toolkit

More than a repository of cancer-related splicing events, ASCancer Atlas also developed a series of visualization and online analysis functions to further investigate the splicing events. To visualize the splicing events, human genome annotation GENCODE v19 (17) was adopted, including sequence information, local splicing switches, transcript structures, isoform composition, and Pfam (18) domain annotations. In addition, a suit of online splicing analysis tools was developed. We built a *Splicing Regulatory Network* by integrating the splicing regulators and corresponding targets, which were all manually curated from the published literatures. *Splicing Regulatory Explorer* was built to interactively explore the potential splicing regulatory mechanism based on TCGA multi-omics evidence. The splicing datasets were processed as previously mentioned, and the matched genetic mutations (SNPs and CNVs) and downstream expression datasets were downloaded and preprocessed from UCSC Xena (19). For each splicing event, Fisher's exact test and Pearson correlation analysis were performed to identify potential genetic lesions and expression factors that may explain the splicing event, respectively. The phenotype datasets that support *Survival Analysis* and *Cancer Staging Analysis* of splicing events were collected from the TCGA pan-cancer (PANCAN) project. Survival analysis and cancer staging analysis were performed by R package survival v3.3.1 (https://cran.r-project.org/web/packages/survival/index.html), survminer v0.4.9 (https://github.com/kassambara/survminer) and ggpubr v0.4.0 (https://mirrors.pku.edu.cn/cran/web/packages/ggpubr/index.html). By integrating drug-gene interactions data from multiple sources, e.g. the Drug–Gene Interaction Database (DGIdb) (20), DrugBank (21), RCSB PDB (22) and BioLiP (23), the ligand-receptor interactions of each drug–gene interaction were obtained. To clarify the impact of different isoforms of the same gene on drug response, the relevant sequences including the binding pocket sequence, target sequence, isoform sequences, and Pfam domain sequences were extracted, and multiple sequence alignment (MSA) was performed by using Clustal Omega provided in the R package msa v1.28.0 (24). The result of MSA was plotted by R package ggmsa v1.2.3 (25). Moreover, the PDBe Molstar (https://github.com/molstar/pdbe-molstar.git) plugin was embedded to interactively view the 3D structure of ligand-receptor interactions. The isoform-specific protein-protein interactions (PPIs) network supporting *Splicing Interaction Analysis* was constructed as described in CanIsoNet (26).

### Database framework and web implementation

ASCancer Atlas was built with Node.js (https://nodejs.org/en/) and deployed in the Centos Linux environment. The back-end database was stored in MongoDB (https://www.mongodb.com/), a free and popular document-based data model. The front-end web user interfaces were developed using Vue.js (https://vuejs.org/). For data visualization, Highcharts (https://www.highcharts.com/) and D3.js (https://d3js.org/) were used to provide interactive charts. Furthermore, the basic data analysis and processing were implemented using R 4.1 (https://www.r-project.org/).

## DATABASE CONTENTS AND USAGE

### Splicing events summary and statistics

ASCancer Atlas aims to provide the most comprehensive cancer-related splicing resources for global researchers. The current version contains two categories of splicing events: CASE and CPSE. ASCancer Atlas houses about 2 million CPSE to characterize the splicing landscapes in human cancers, covering 1 111 805 splicing events of 33 TCGA cancer types and 867 164 splicing events of 31 GTEx normal tissues. Moreover, it also contains 46 591 cancer-specific splicing events. All CPSE are publicly accessible at https://ngdc.cncb.ac.cn/ascancer/download and can be freely downloaded for further analysis.

More importantly and distinctively, ASCancer Atlas built the first repository of experimentally validated CASE with established oncogenic roles summarized from extensive functional studies. By manually curating 610 published literatures, a total of 2006 high-confidence CASE with detailed information were archived, involving 29 cancer primary sites and 60 human cancer subtypes. For each CASE, ASCancer Atlas records the complete information of upstream splicing regulators, splicing event annotations, main downstream oncogenic effects and potential treatment targets. This layered structure may help users to rapidly capture the functional axis of aberrant splicing of their interest, quickly design experimental validations in their own research cases, and compare their unique findings in the context of previous research discoveries.

### User-friendly web interfaces for browsing, retrieving and visualizing splicing events

**Figure 2.**
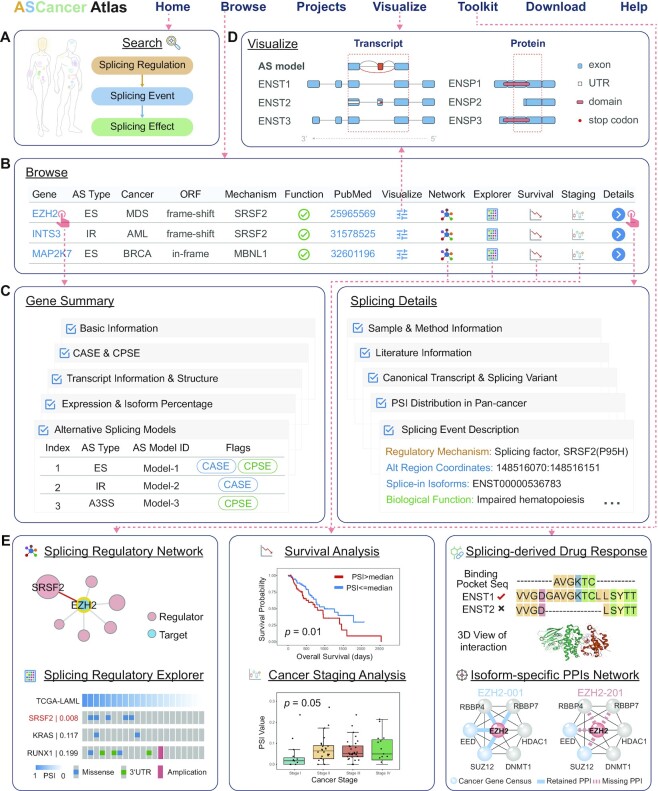
Thumbnails of web interfaces in ASCancer Atlas. (**A**) Quick search function provided in the ‘Home’ page. (**B**) CASE or CPSE list, as well as visualization and analysis hyperlinks provided in ‘Browse’ page. (**C**) ‘Gene Summary’ and ‘Splicing Details’ pages show the full-scale metadata of each gene and splicing event. (**D**) Interactively visualize splicing events at the local genomic region, the whole transcript and protein levels. (**E**) A suit of online splicing analysis tools equipped in ASCancer Atlas, including *Splicing Regulatory Network*, *Splicing Regulatory Explorer*, *Survival Analysis*, *Cancer Staging Analysis*, *Splicing-derived Drug Response* and *Isoform-specific PPIs Network*. Abbreviations: AS: alternative splicing. UTR: untranslated region. MDS: myelodysplastic syndromes. AML: acute myeloid leukemia. BRCA: breast invasive carcinoma. PSI: percent spliced in.

As shown in Figure 2, ASCancer Atlas designed a series of user-friendly web interfaces that enable users to easily browse, retrieve and visualize splicing events. The ‘Home’ page provides a comprehensive overview, where users can freely navigate by the quick search function which provided inquiries of splicing regulator, gene name, cancer type or splicing effect (Figure 2A). Users can also directly browse and inquire the CASE or CPSE according to the advanced search function provided by the ‘Browse’ page. In addition, this page provides additional hyperlinks to *Gene Summary*, *Splicing Details*, *Splicing Visualization*, and *Splicing Toolkit* pages (Figure 2B). *Gene Summary* page shows the detailed information of gene, including *Basic Information, Gene Expression and Isoform Percentage in Pan-Cancer, Transcript Information and Structure, Alternative Splicing Models, CASE* and *CPSE. Splicing Details* page provides the detailed curation information of each CASE, including *Splicing Event Description*, *Sample & Method Information*, *Literature Information*, *PSI distribution in Pan-Cancer*, *Canonical Transcript* and *Splicing Variant* (Figure 2C). *Splicing Visualization* page provides interactive visualization of all collected CASE and CPSE of a single gene, and shows the PSI distribution of each event in TCGA pan-cancer. Users can also directly view the splicing changes at the local genomic region, the whole transcript and protein levels (Figure 2D).

### Interactive splicing analysis toolkit

ASCancer Atlas has an online splicing analysis toolkit to further explore the cancer splicing in multiple aspects, involving potential regulators discovery (*Splicing Regulatory Network* and *Splicing Regulatory Explorer*), clinical relevance (*Survival Analysis* and *Cancer Staging Analysis*) and treatment suggestion (*Splicing-derived Drug Response* and *Isoform-specific PPIs Network*) (Figure 2E).

#### Splicing regulatory network

A recent study suggested that splicing dysregulation tends to exert physiological functions in the form of coordinated splicing networks, not a single event (27). Splicing disorders in cancers are further complicated by the multi-maps between splicing targets and *trans-*acting splicing factors, as well as internal regulations between the splicing factors. ASCancer Atlas provides so far the most complete mis-splicing regulatory network in human cancers, which attempts to link all cancer-associated splicing events from individual studies together in a global view for a better understanding of the coordinated effects of splicing dysregulation (Supplementary Figure S1). Users can interactively view their interested splicing regulators or the corresponding target genes from a systematic and global perspective.

#### Splicing regulatory explorer

Alterations in *trans*-acting splicing regulatory factors accounts for most splicing dysregulation in human cancers, especially in hematological malignancies (28). ASCancer Atlas developed an online tool to uncover the potential splicing regulators based on TCGA multi-omics evidence. At present, the tool supports the exploration of potential splicing regulators from the levels of genetic variants (SNVs/CNVs) and transcriptional expressions. Briefly, with selected event and cancer type, the tool provides an interactive mutation oncoprint and expression heatmap with samples (columns) sorted by the splicing intensity, together with statistics from the pre-ranked enrichment test. For each AS event, the top 10 potential regulators are presented by default and users can also manually check customized genes. In addition, the tool can also help users to view the correlation between gene expression and PSI of AS events. This tool was designed to reveal the full spectrum and possible synergistic combinations of splicing regulatory mechanisms. Likewise, this function would help users to target potential regulatory candidates and design experiments to test any causal associations.

#### Survival analysis and cancer staging analysis

Survival analysis allows users, especially clinicians, to rapidly estimate the prognostic significance of a given splicing event in the specified cancer type. This may assist them to focus on the candidate splicing events for constructing the prognosis prediction model, and ultimately for cancer treatment. It was reported that AS plays a critical role in developmental processes and is temporally regulated precisely (29). Therefore, the cancer staging analysis tool was developed in ASCancer Atlas to help users to view the sequential dynamics of a given splicing event at different pathological stages of tumors.

#### Splicing-derived drug response

AS in cancers may cause therapeutic resistance by changing the binding affinity of targeting agents (30,31). This tool allows users to explore the impact of AS on drug-target interactions at the isoform level. Users can investigate the perturbation of different isoforms of the same gene on the drug binding interface by entering the name of gene or drug of interest. In addition, users can also interactively manipulate the 3D structure of receptor-ligand interactions and view the result of multiple sequence alignment for each receptor–ligand interaction to explore how AS affects drug-gene interactions and leads to drug resistance. This tool would help to develop new therapeutic strategies to overcome the splicing-derived drug resistance.

#### Isoform-specific PPIs network

Splicing dysregulation in cancers may lead to the emergence of cancer-specific isoform switches, and thereby affecting PPIs. This tool aims to explore the functional roles of alternative splicing on PPIs at the isoform level. Users can clearly view the missing or retained isoform-specific PPIs network by entering gene or transcript ID of interest. Users can also set the maximum number of PPIs for visualization.

### Demonstrations of ASCancer atlas usage

**Figure 3.**
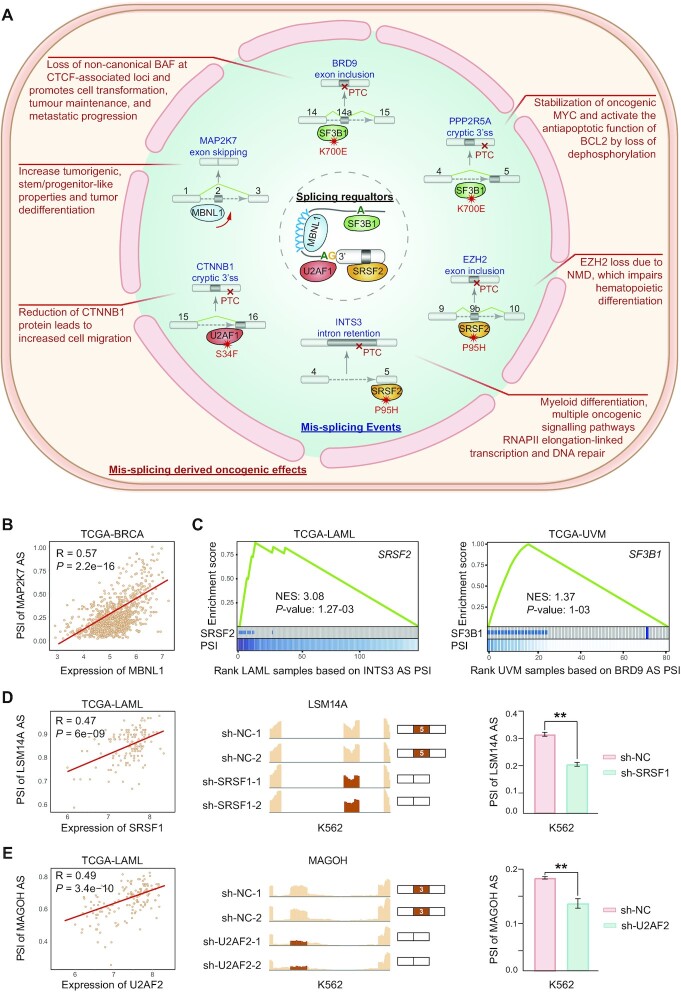
Demonstrations of ASCancer Atlas usage by case studies. (**A**) Examples of known splicing dysregulation in human cancers including carcinogenic mis-splicing events, their regulatory mechanism and oncogenic effects. (**B**) Correlation between *MBNL1* expression and PSI values of *MAP2K7* AS in the TCGA-BRCA cohort. Analysis done by the online tool *Splicing Regulatory Explorer* of ASCancer Atlas. (**C**) *SRSF2* mutation oncoprint with columns sorted by the splicing intensity of *INTS3* AS in TCGA-LAML (left). *SF3B1* mutation oncoprint with columns sorted by the splicing intensity of *BRD9* AS in TCGA-UVM (right). *P*-values were calculated from pre-ranked enrichment test. Analysis done by the online tool *Splicing Regulatory Explorer* of ASCancer Atlas. (**D**) Identification of *LSM14A* cassette exon 5 skipping event in the TCGA-LAML cohort and validation of putative *SRSF1* regulator by knockdown experiments in human K562 leukemia cell lines. Correlation between *SRSF1* expression and event PSI in the TCGA-LAML cohort, analysis done by *Splicing Regulatory Explorer* (left), exon 5 inclusion preference after knocking down *SRSF1* in K562 (middle), and splicing changes between *SRSF1* knockdowns and counterparts in K562, *P*-value was calculated from t-test (right). (**E**) Identification of *MAGOH* cassette exon 3 skipping event in the TCGA-LAML cohort and validation of putative *U2AF2* regulator by knockdown experiments in human K562 leukemia cell lines. Correlation between *U2AF2* expression and event PSI in the TCGA-LAML cohort, analysis done by *Splicing Regulatory Explorer* (left), exon 3 inclusion preference after knocking down *U2AF2* in K562 (middle), and splicing changes between *U2AF2* knockdowns and counterparts in K562, *P*-value was calculated from t-test (right). Abbreviations: NES: normalized enrichment score. AS: alternative splicing. PTC: premature termination codon. NMD: nonsense-mediated decay. BRCA: breast invasive carcinoma. LAML: acute myeloid leukemia. UVM: uveal melanoma. PSI: percent spliced in.

ASCancer Atlas has a wide range of application scenarios, and one key task in cancer-associated splicing studies is to find the correct regulator of functional mis-splicing events in cancers. Here, we demonstrate how to achieve this by ASCancer Atlas. We first test on a number of CASE with known regulators as positive controls. Decades of efforts have indicated that frequently altered splicing factors are key drivers of malignant tumors, especially in hematological malignancies (8). As show in Figure 3A, studies indicated that *SRSF2-*P95H mutation leads to aberrant splicing of *EZH2* and *INTS3* in acute myeloid leukemia (AML). These aberrant splicing introduce a premature termination codon (PTC) expected to cause nonsense-mediated decay and degradation of mis-spliced mRNA, which leads to impaired hematopoietic differentiation (32,33). Moreover, Liu *et al.* found that *SF3B1-*K700E mutation induces cryptic 3’ splice site usage in one protein phosphatase 2A subunit *PPP2R5A*, which results in *MYC* and *BCL2* activations (34). Angela *et al.* also revealed that abnormal splicing of *CTNNB1* is caused by *U2AF1-*S34F mutation (35). In addition, not only mutations in splicing factors, but also altered expression level may cause carcinogenic mis-splicing. For example, down-regulation of splicing regulator *MBNL1* increases exon 2 skipping of *MAP2K7*, which increases cancer stemness and drives tumor dedifferentiation (36). All these well-studied dysregulation of CASE above can be perfectly reproduced by our online analysis feature: *Splicing Regulatory Explorer* from the toolkit module of ASCancer Atlas (Figure 3B, C, Supplementary Figure S2).

Next, in addition to successfully capture known splicing dysregulation of CASE, ASCancer Atlas is also capable of predicting unknown regulatory factors of CPSE. Following the above knowledge that splicing aberrations could result from expression changes of splicing factors, we identified AS events, whose PSI value is significantly correlated with the expression of splicing factors by *Splicing Regulatory Explorer* function. Specifically, in the TCGA-LAML cohort, we found that exon 5 skipping of *LSM14A* is positively correlated with *SRSF1* expression, while enhanced *U2AF2* expression is associated with exon 3 skipping of *MAGOH*. To verify these findings, we downloaded the RNA-Seq data from the knockdown experiments using human K562 cells from the ENCODE (37) database. Strikingly, the splicing changes from these knockdown experiments are highly consistent with our findings by ASCancer Atlas. The exon 5 inclusion level of *LSM14A* is significantly reduced when knocking down *SRSF1* (Figure 3D), the exon 3 of *MAGOH* is more skipped after knocking down *U2AF2* (Figure 3E), which indicates that *SRSF1* and *U2AF2* could be the real regulators of these splicing events, respectively. More such evidences can be found in Supplementary Figure S2.

## CONCLUDING REMARKS AND FUTURE DEVELOPMENT

Ever-increasing evidences have suggested that pre-mRNA mis-splicing can fuel the initiation, progression or drug resistance of cancers. To the best of our knowledge, ASCancer Atlas is the first knowledgebase of carcinogenic AS in human cancers, which provides a comprehensive resource including CASE, CPSE, interactive splicing visualization and multi-scale online splicing analysis toolkit. Different from the existing data-oriented splicing databases, ASCancer Atlas has unique strengths as the following: (i) it collects a total of 2006 high-confidence CASE, and each CASE features a standardized curation model. The regulatory mechanisms and determined oncogenic splicing effects have been verified through functional experiments, which can assist researchers to rapidly capture the functional axis for experimental testing; (ii) by connecting all CASE and their regulators from hundreds of publications, it provides the most complete and systematic mis-splicing regulatory networks in human cancers, enabling a better understanding of the coordinated effects of splicing disorders; (iii) equipped with a suit of online splicing analysis tools, which facilitate the exploration of splicing events in multiple dimensions and contribute to the identification of potential regulators, clinically relevant events, and possible therapeutic targets; (iv) the concept of website design follows a concise logic axis of standard study on cancer splicing from splicing regulator, mis-splicing events, oncogenic effects to therapeutic interventions. The emphasis of this layered web organization aims to better serve and meet the requirements of the different components of a conventional study on aberrant splicing in cancers.

As one of the many resources of the National Genomics Data Center (38), ASCancer Atlas will be devoted to serving as an open-access and comprehensive one-stop platform for AS studies. Future directions include regular curation of newly reported CASE every three months, continuous expansion of CPSE every six months, and exploratory development of handy online splicing tools. For any new-added CPSE, ASCancer Atlas will strictly follow the same computational pipeline as the pan-cancer atlas splicing project and perform quality control strategies to reduce the possible batch effect. Additionally, given that tumors contain more neo-junctions than normal samples, we intend to provide a new analysis tool of ‘putative splicing-derived neoantigens’ for cancer immunotherapy by predicting the major histocompatibility complex binding ability of tumor-specific splicing isoforms. With the ever-increase amount of long-read RNA sequencing data of normal and malignant tissues, we will consider building an independent module in the next release to store the splicing resources identified from the third-generation sequencing data. Due to the important regulation roles of epigenetic factors in alternative splicing, we will add DNA methylation factors to *Splicing Regulatory Explorer* tool by establishing connections with related databases, such as MethBank (39) and EWAS Open Platform (40). In all, we believe that ASCancer Atlas will attract considerable interest in a wide range of audiences including cancer biologists, molecular biologists, computational biologists and clinical oncologists.

## DATA AVAILABILITY

ASCancer Atlas is freely available online at https://ngdc.cncb.ac.cn/ascancer. Users can directly download the splicing resources without registration or login.

## Supplementary Material

gkac955_Supplemental_File
