# Supplementary material for: ASCancer Atlas: a comprehensive knowledgebase of alternative splicing in human cancers
Source: Nucleic Acids Res. 2022 Nov 1;51(D1):D1196–204. doi: 10.1093/nar/gkac955 (PMC9825479; doi:10.1093/nar/gkac955)
Supplement: gkac955_Supplemental_File [file gkac955_supplemental_file.pdf]

Supplementary Figures

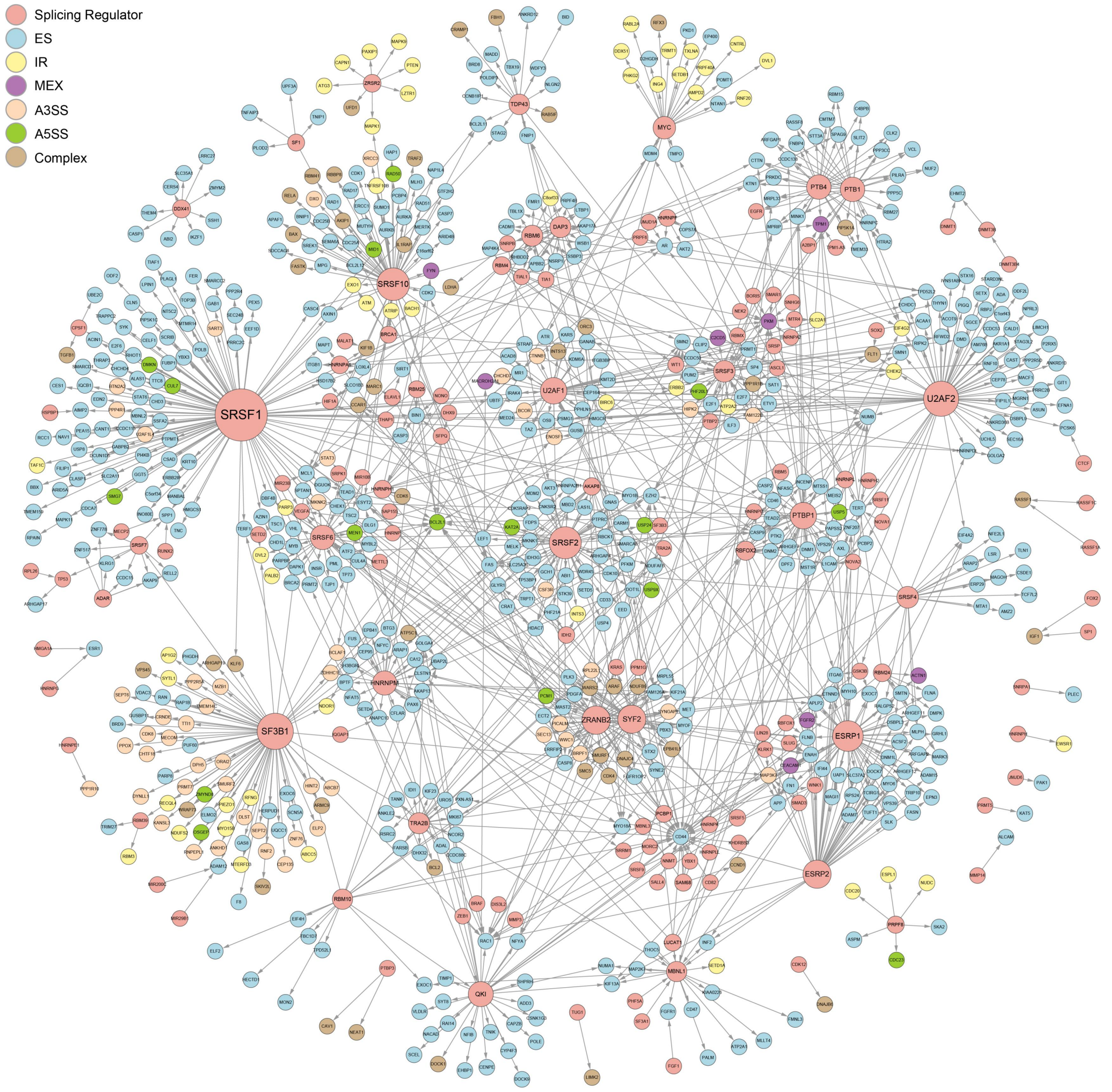

Supplementary Figure S1. The Splicing regulatory network built in ASCancer Atlas. The splicing regulatory network was constructed by linking splicing regulators (as source nodes) and corresponding AS genes (as target nodes), which were all curated from published studies. Nodes of different colors represent different AS types. Node size of splicing regulator is proportional to the number of targets. Abbreviations: ES: exon skipping. IR: Intron retention. A3SS: alternative 3′ splice site. A5SS: alternative 5′ splice site. MEX: mutually exclusive exons.

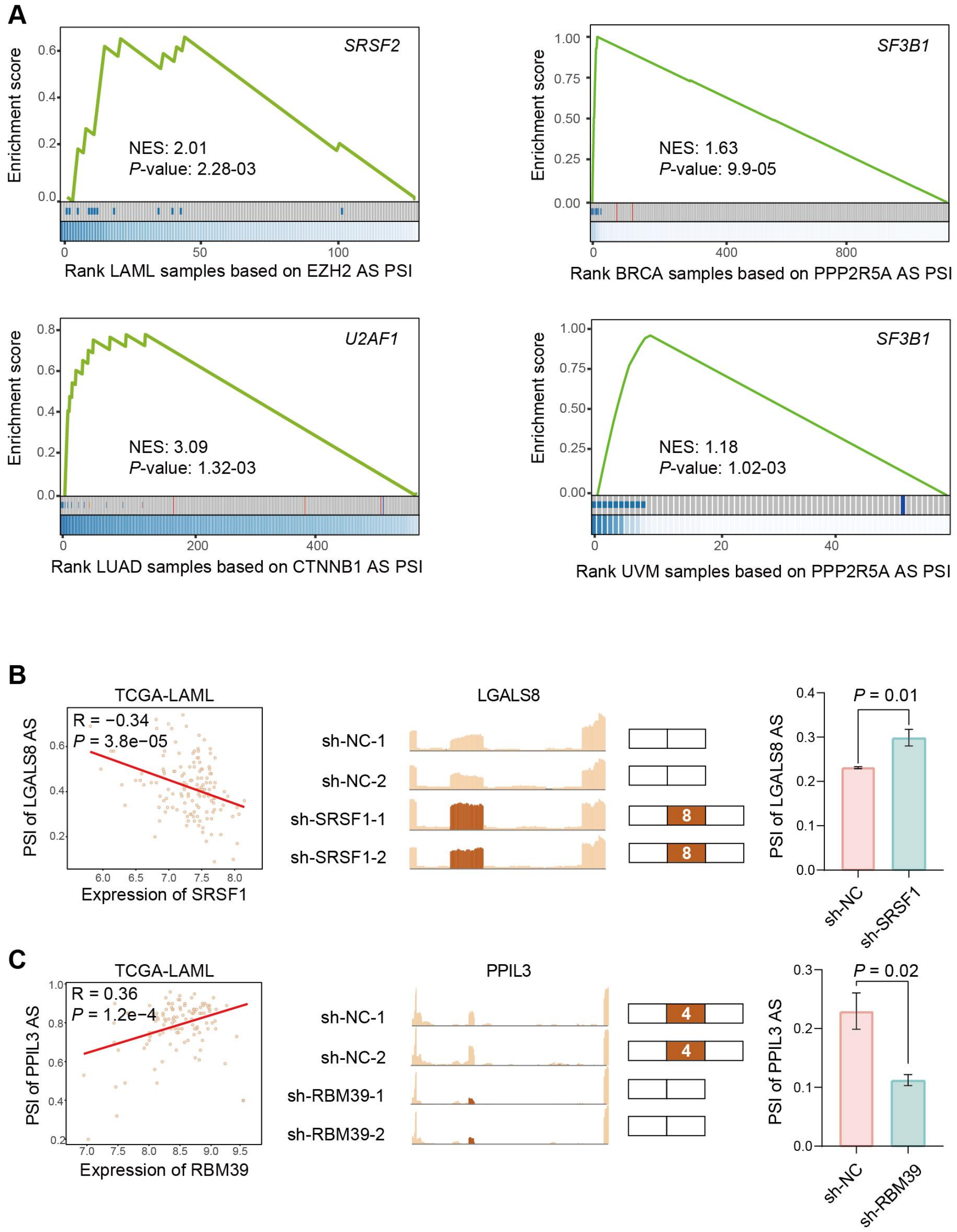

Supplementary Figure S2. (A) *SRSF2* mutation oncoprint with columns sorted by the splicing intensity of *EZH2* AS in TCGA-LAML (upper-left). *SF3B1* mutation oncoprint with columns sorted by the splicing intensity of *PPP2R5A* AS in TCGA-BRCA (upper-right). *U2AF1* mutation oncoprint with columns sorted by the splicing intensity of *CTNNB1* AS in TCGA-LUAD (lower-left). *SF3B1* mutation oncoprint with columns sorted by the splicing intensity of *PPP2R5A* AS in TCGA-UVM (lower-right). *P*-values were calculated from pre-ranked enrichment test.

Analysis done by the online tool *Splicing Regulatory Explorer* of ASCancer Atlas. **(B)** Identification of *LGALS8* cassette exon 8 skipping event in the TCGA-LAML cohort and validation of putative *SRSF1* regulator by knockdown experiments in human K562 leukemia cell lines. Correlation between *SRSF1* expression and event PSI in the TCGA-LAML cohort, analysis done by *Splicing Regulatory Explorer* (left), exon 8 inclusion preference after knocking down *SRSF1* in K562 (middle), and splicing changes between *SRSF1* knockdowns and counterparts in K562, *p*-value was calculated from t-test (right). **(C)** Identification of *PPIL3* cassette exon 4 skipping event in the TCGA-LAML cohort and validation of putative *RBM39* regulator by knockdown experiments in human K562 leukemia cell lines. Correlation between *RBM39* expression and event PSI in the TCGA-LAML cohort, analysis done by *Splicing Regulatory Explorer* (left), exon 4 inclusion preference after knocking down *RBM39* in K562 (middle), and splicing changes between *RBM39* knockdowns and counterparts in K562, *p*-value was calculated from t-test (right). Abbreviations: NES: normalized enrichment score. AS: alternative splicing. AML: acute myeloid leukemia. BRCA: breast invasive carcinoma. LUAD: lung adenocarcinoma. UVM: uveal melanoma. PSI: percent spliced in.
